# Supplementary material for: A Novel Ruthenium(II) Polypyridyl Complex Bearing 1,8-Naphthyridine as a High Selectivity and Sensitivity Fluorescent Chemosensor for Cu2+ and Fe3+ Ions
Source: Molecules. 2019 Nov 7;24(22):4032. doi: 10.3390/molecules24224032 (PMC6891798; doi:10.3390/molecules24224032)
Supplement: Supplementary file 1 [file molecules-24-04032-s001.pdf]

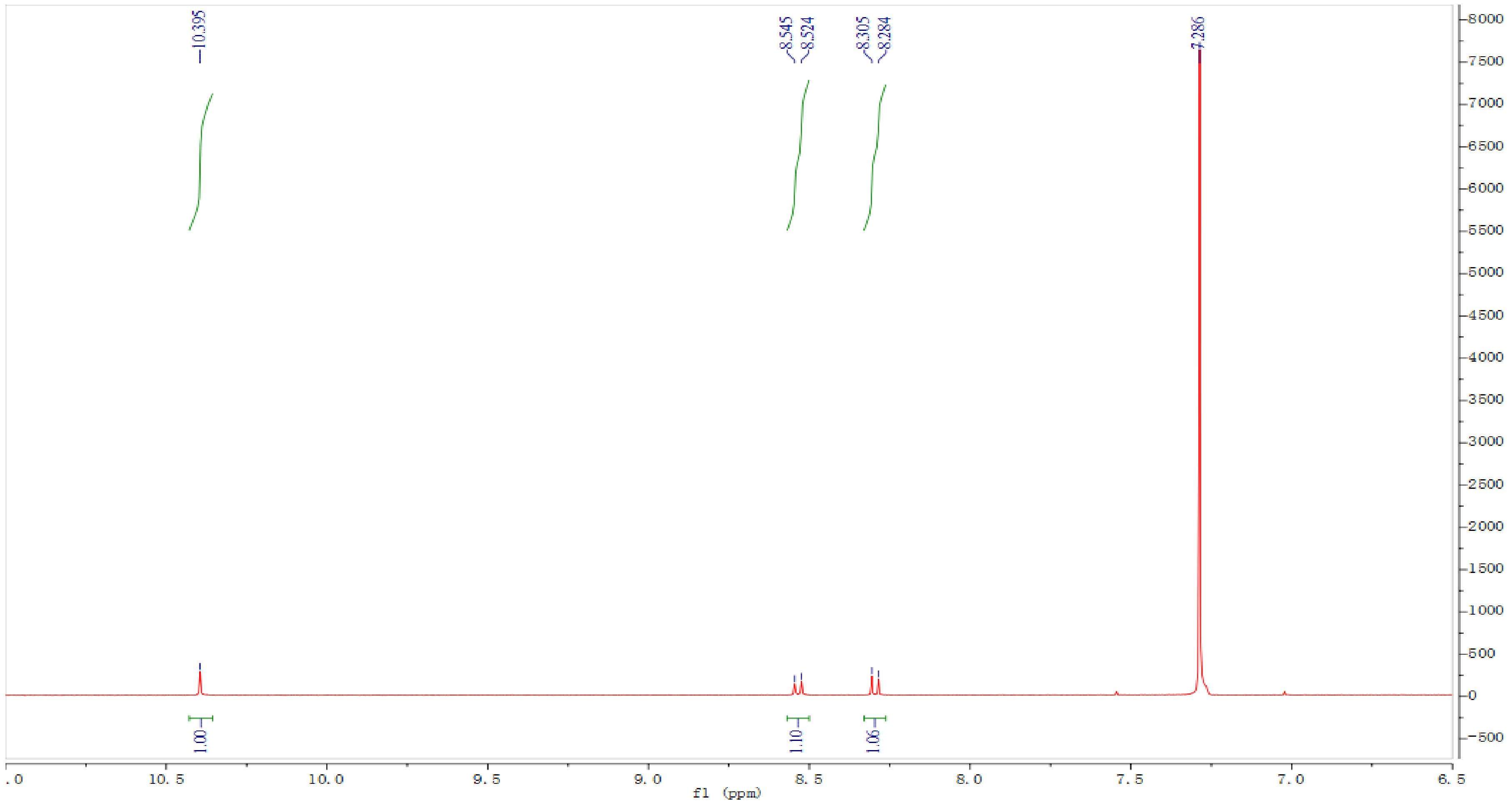

Figure S 1. ^{1}H NMR spectrum for 1,8-naphthyridine-2,7-dicarbaldehyde (in $CDCl_3$).

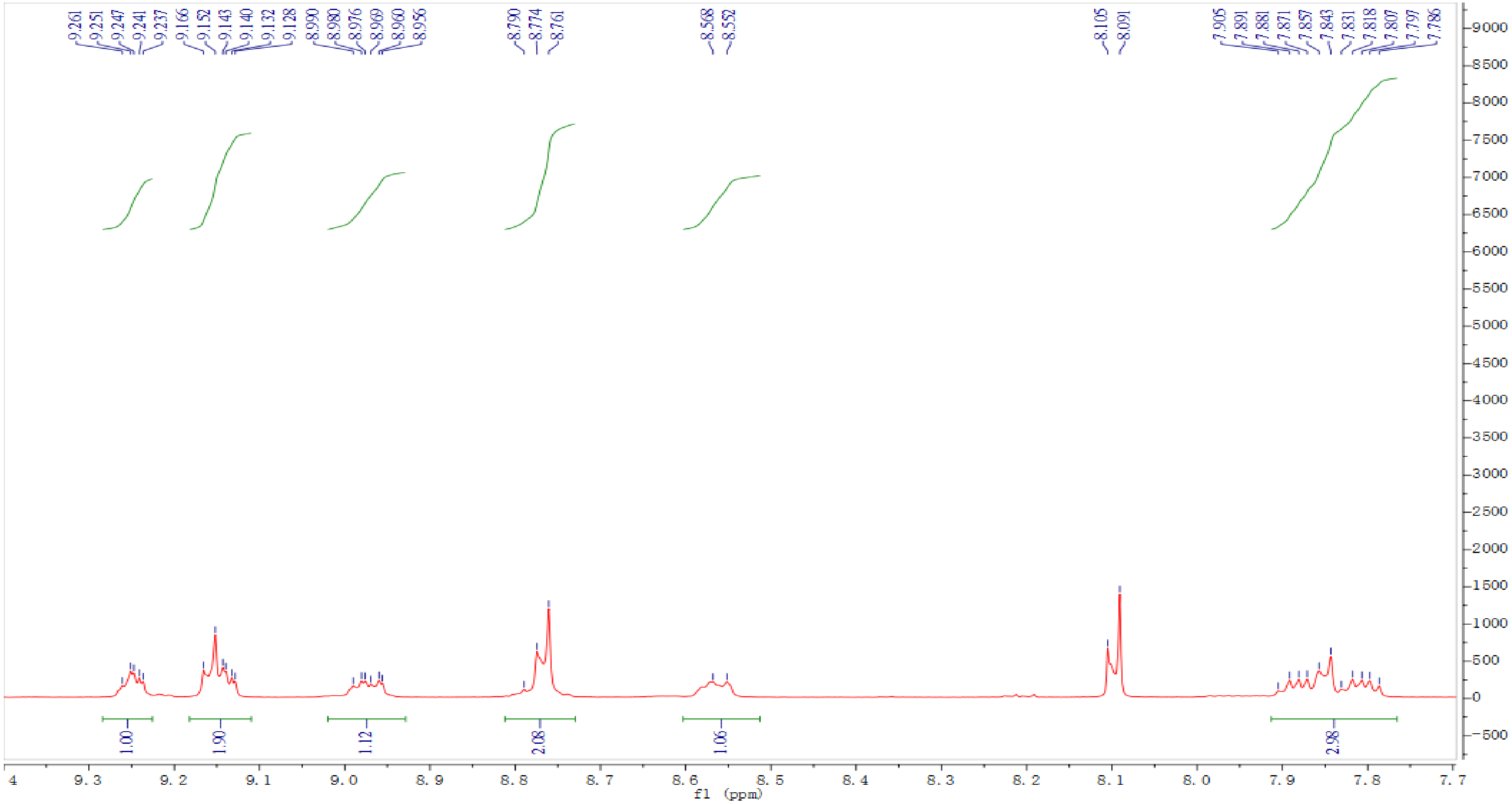

Figure S 2. ^{1}H NMR spectrum for L (in DMSO-d^6 and a small amount of $CDCl_3$).

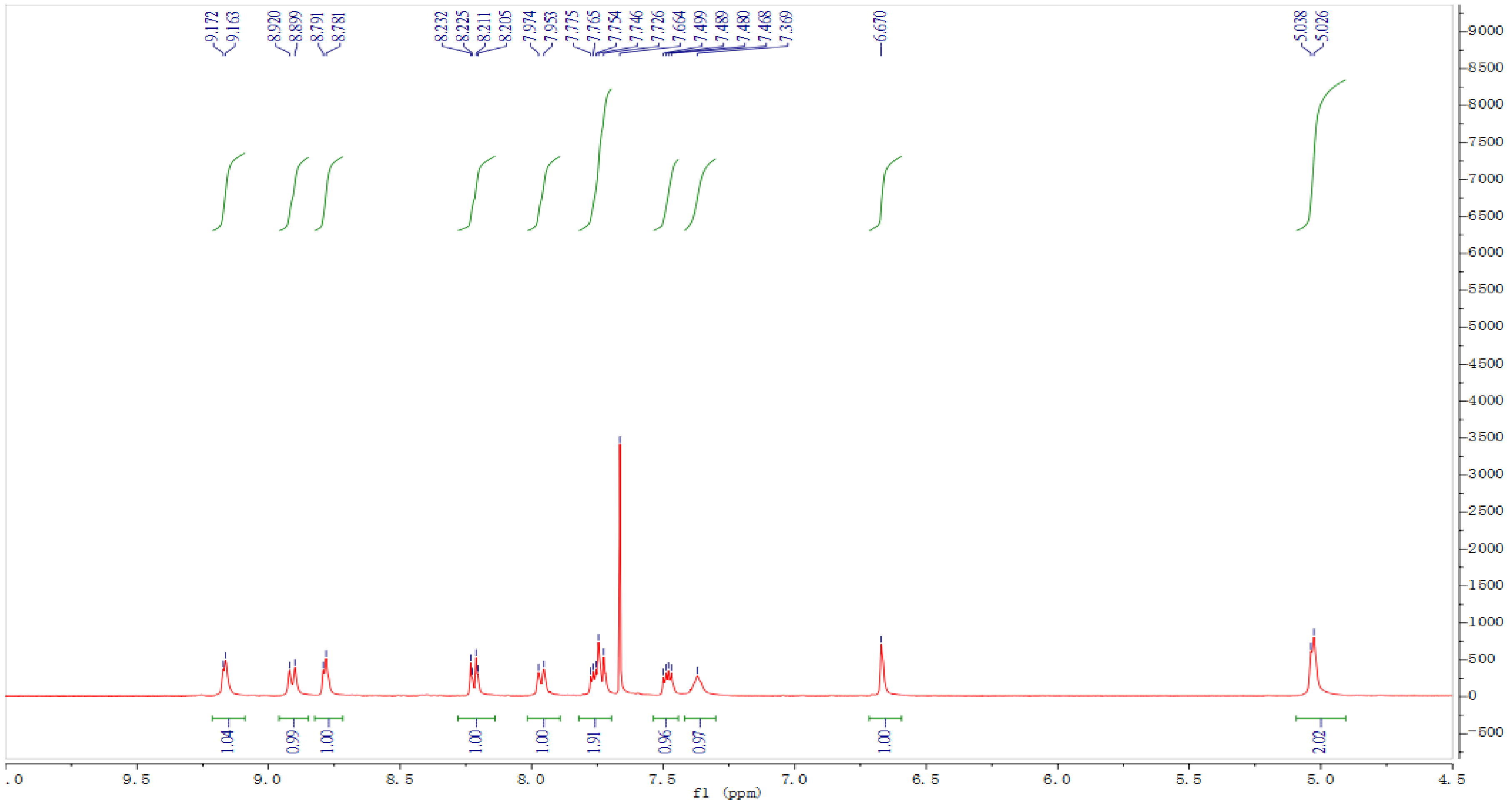

Figure S 3. ^{1}H NMR spectrum for H_2L (in DMSO-d^6 and a small amount of $CDCl_3$).

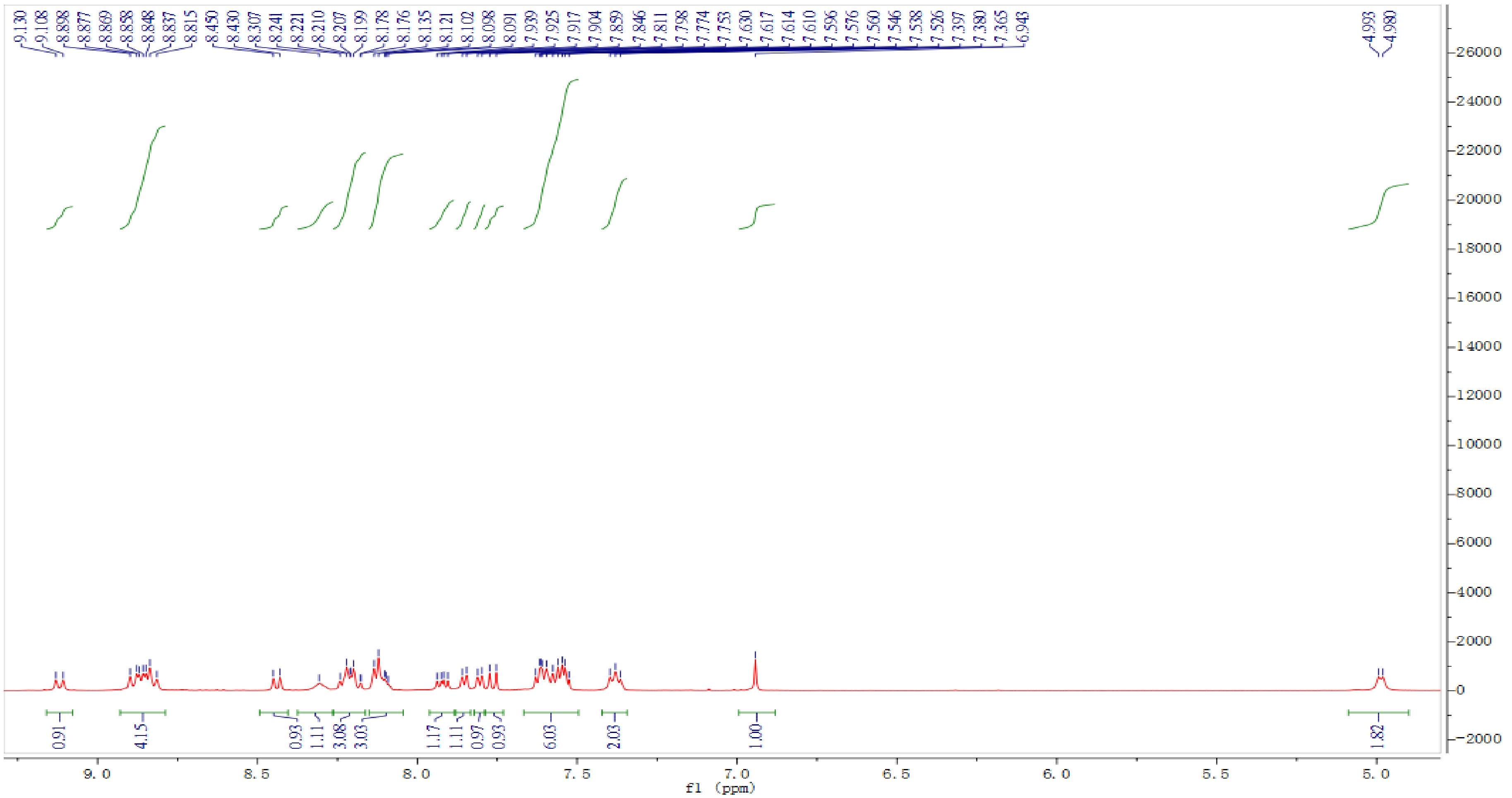

Figure S 4. ^{1}H NMR spectrum for $[\{Ru(bpy)_2\}_2(\mu_2\text{-}H_2L)](PF_6)_4$ (in DMSO-d^6).

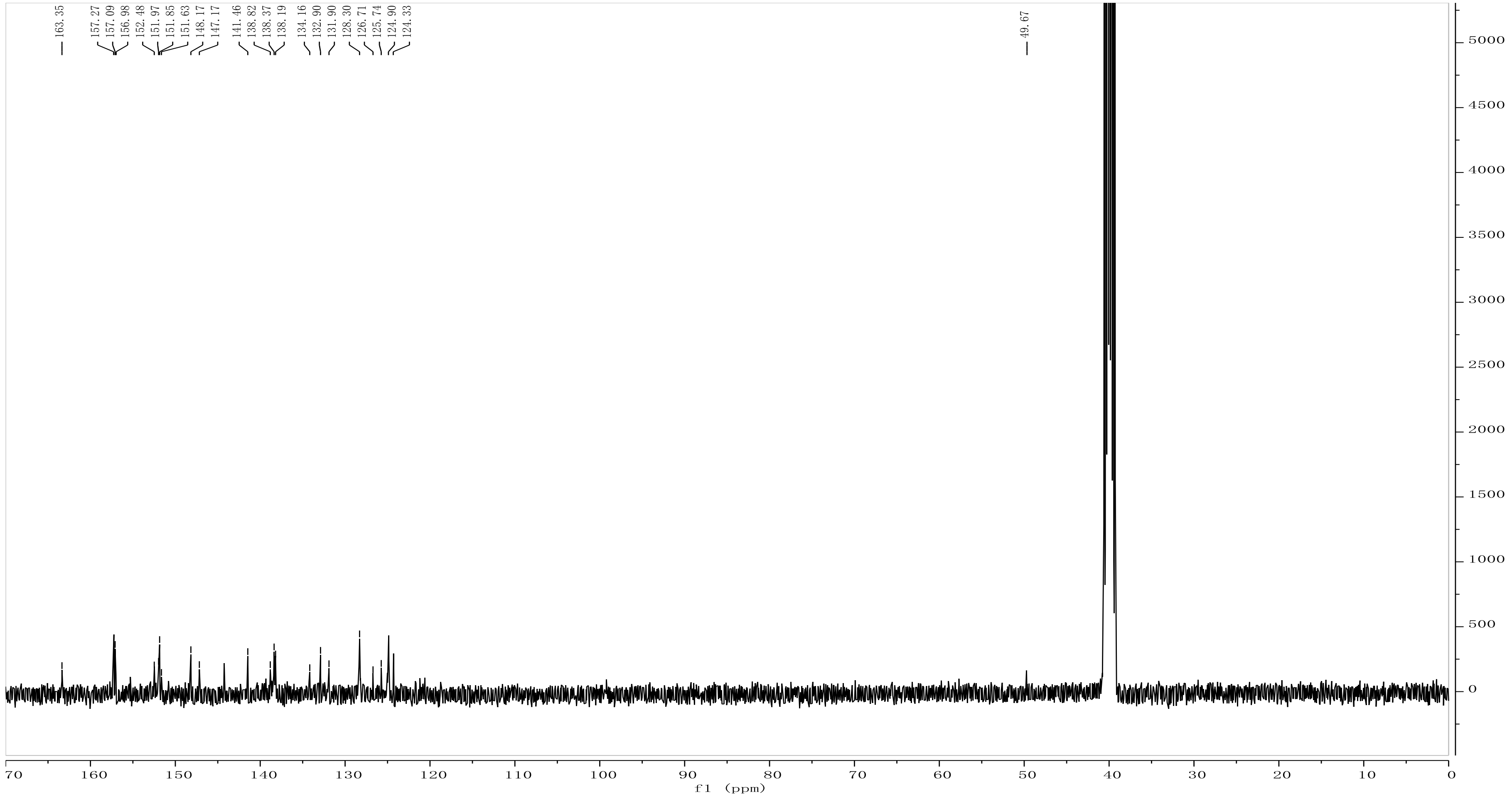

Figure S 5 ^{13}C NMR spectrum for [{Ru(bpy)$_2$}$_2$(μ_2-H$_2$L)](PF$_6$)$_4$ (in DMSO-d^6).

Generic Display Report

Analysis Info		Acquisition Date	9/19/2019 6:07:12 AM
Analysis Name	D:\Data\hechixian\BASE.d		
Method	DEF_MS.M	Operator	BDAL
Sample Name	1	Instrument	amaZon SL
Comment			

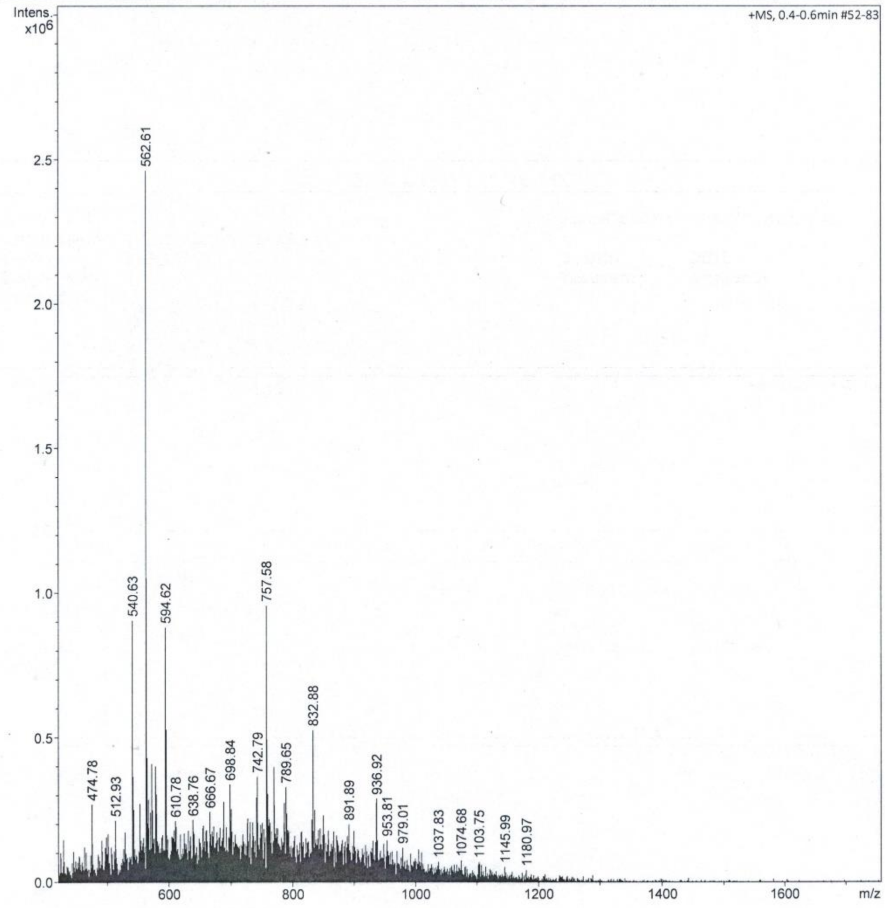

Bruker Compass DataAnalysis 4.1 printed: 9/19/2019 6:08:37 AM by: BDAL Page 1 of 1

Figure S 6. ESI-MS for L.

Generic Display Report

Analysis Info

Analysis Name	D:\Data\hechixian\PEITI.d	Acquisition Date	9/19/2019 5:55:57 AM
Method	DEF_MS.M	Operator	BDAL
Sample Name	Internal errorr: String ID=101 is not defined in Instrument	Instrument	amaZon SL
Comment	Controller resource. The intended message could not be displayed.		

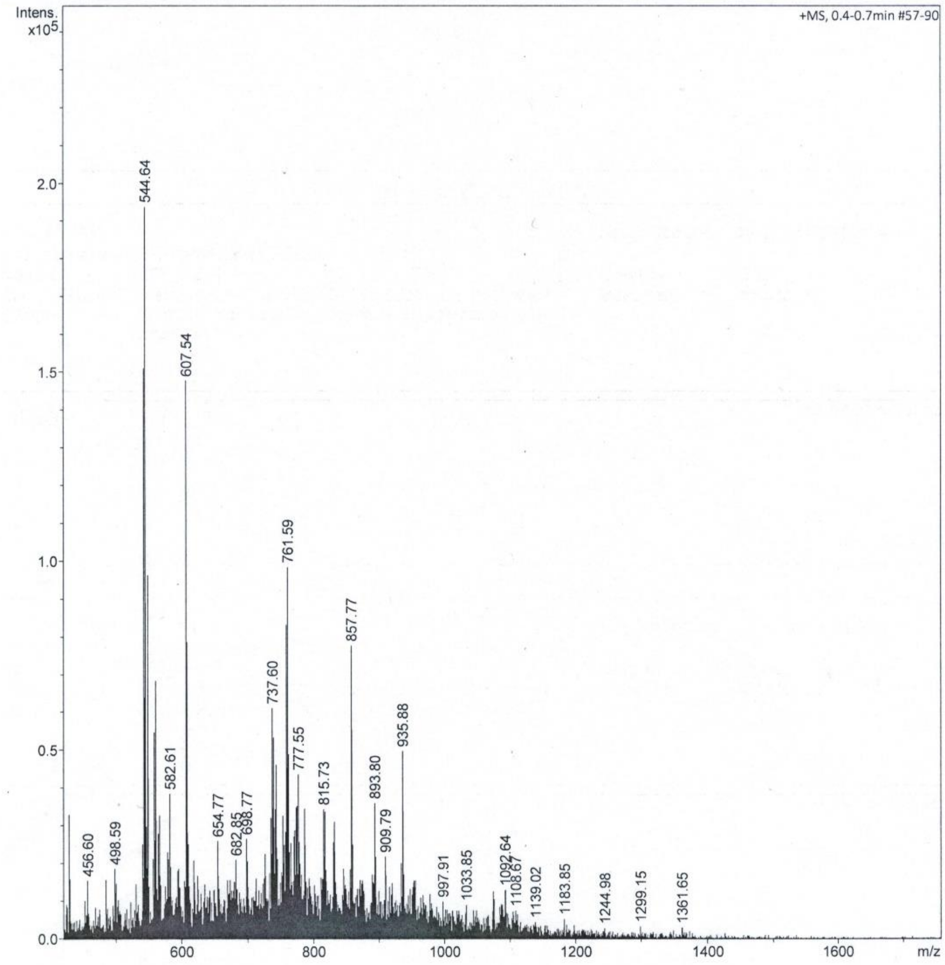

Bruker Compass DataAnalysis 4.1 printed: 9/19/2019 6:05:39 AM by: BDAL Page 1 of 1

Figure S 7. ESI-MS for H_2L.

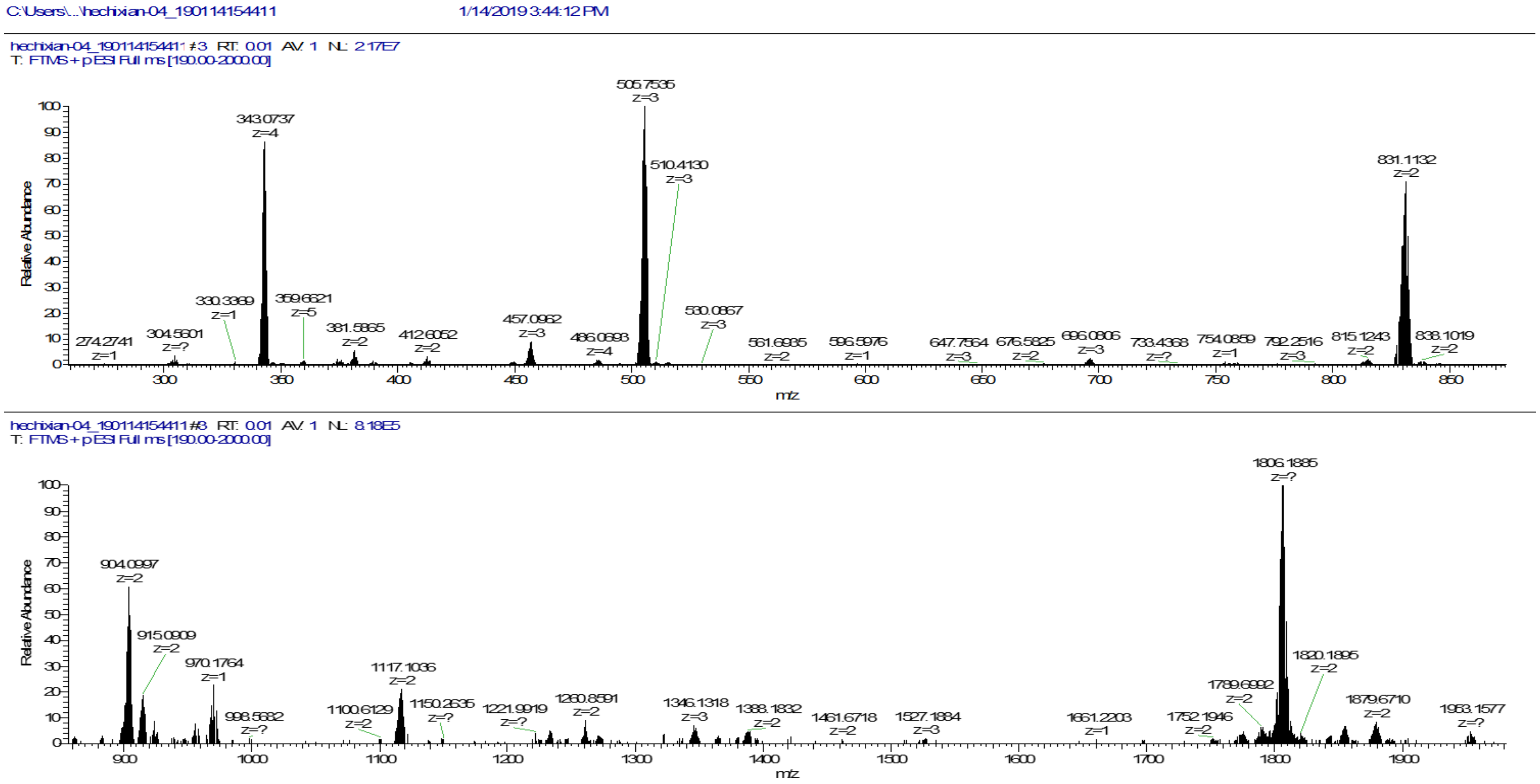

Figure S 8. ESI-HRMS for $[\{Ru(bpy)_2\}_2(\mu_2\text{-}H_2L)](PF_6)_4$

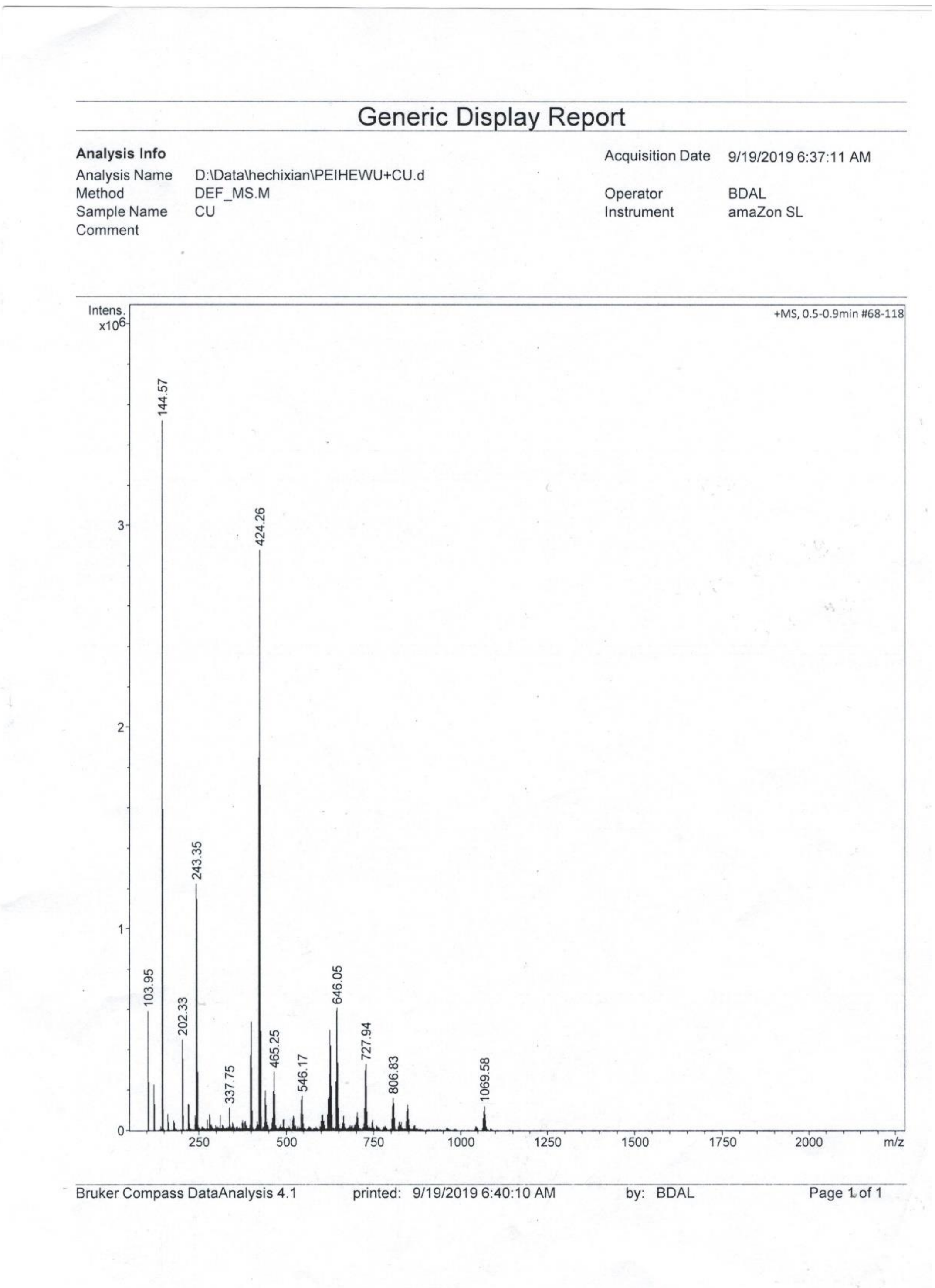

Figure S 9. ESI-MS for $[(\{Ru(bpy)_2\}_2(\mu_2\text{-}H_2L))_3Cu_2](PF_6)_{12}(ClO_4)_4$

Generic Display Report

Analysis Info

Analysis Name	D:\Data\hechixian\PEIHEWU+FE.d	Acquisition Date	9/19/2019 6:23:09 AM
Method	DEF_MS.M	Operator	BDAL
Sample Name	FE	Instrument	amaZon SL
Comment			

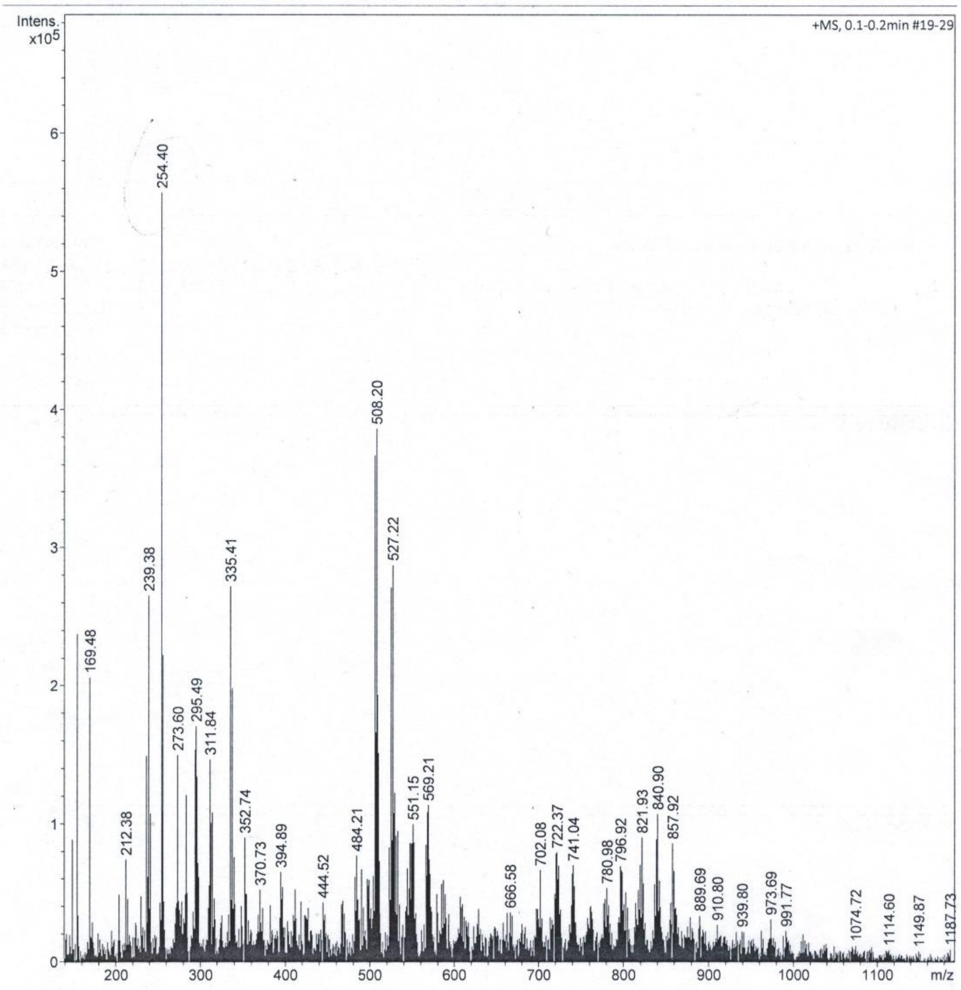

Bruker Compass DataAnalysis 4.1 printed: 9/19/2019 6:38:00 AM by: BDAL Page 1 of 1

Figure S 10. ESI-MS for $[(\{Ru(bpy)_2\}_2(\mu_2\text{-}H_2L))Fe](PF_6)_4(ClO_4)_3$

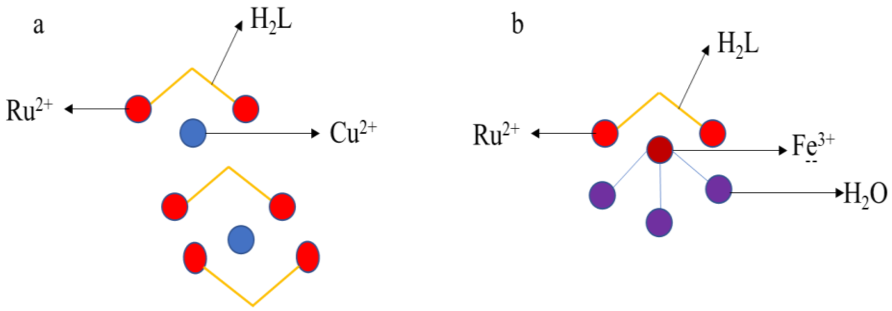

Figure S 11. The “sandwich” of hypothetical structures of new product of Cu^{2+}(a) and hypothetical structures of new product of Fe^{3+}(b)
